# *Malassezia pachydermatis* Carriage in Dog Owners

**DOI:** 10.3201/eid1101.040882

**Published:** 2005-01

**Authors:** Daniel O. Morris, Kathleen O’Shea, Frances S. Shofer, Shelley Rankin

**Affiliations:** * University of Pennsylvania, Philadelphia, Pennsylvania, USA; *Malassezia pachydermatis* Carriage in Dog Owners

**Keywords:** Dogs, Dermatitis, atopic, Yeasts, Malassezia, Fungemia, Intensive care, neonatal, Zoonoses, Research

## Abstract

Yeasts of the genus *Malassezia* serve as both commensal microorganisms and pathogens on the skin of humans and domestic animals. Although rare, cases of life-threatening fungemia in people have been attributed to *Malassezia pachydermatis*, for which dogs are a natural host. Zoonotic transfer has been documented from dogs to immunocompromised patients by healthcare workers who own dogs. We investigated the role of pet dogs as risk factors for mechanical carriage of *M. pachydermatis* on human hands. Dogs and their owners were sampled as pairs, by fungal culture and nested polymerase chain reaction (PCR). Although fungal culture was not a reliable means by which to detect carriage of the yeast on human hands, PCR identified *M. pachydermatis* on most (≈93%) human participants. Human carriage of ubiquitous opportunistic pathogens such as *M. pachydermatis* underscores the importance of good hand hygiene by healthcare professionals.

## Abstract 2

*Malassezia pachydermatis* is commonly carried on the hands of dog owners and may cause disease in immunocompromised persons.

Yeasts of the genus *Malassezia*, part of the normal cutaneous microflora of mammals, can cause life-threatening fungemia and other nosocomial infections in immunocompromised humans, especially in preterm neonates (*1*–*3*). While disease in humans is most commonly caused by *Malassezia furfur*, a commensal of human skin (*4*), it has also resulted from *M. pachydermatis*, for which dogs are a natural host (*5*–*8*). In some cases, the sources of human infections have been traced to pet dogs owned by healthcare workers (*9*).

In normal dogs with healthy skin, *M. pachydermatis* colonizes the stratum corneum in very low numbers (*10*). In dogs with allergic skin disease, however, the numbers of *M. pachydermatis* may increase dramatically on the skin and within the ear canals (*11*–*13*). The potential for human exposure to the organism is therefore quite great. While no evidence has shown that dogs represent an overt health concern to immunocompromised humans, the increasing incidence of immune suppression in humans worldwide suggests that a survey of the zoonotic potential of this organism is relevant to modern hospital hygiene practices.

We hypothesized that mechanical transfer of *M. pachydermatis* from the inflamed skin of dogs with *M. pachydermatis* infection to the healthy skin of humans occurs commonly. We also hypothesized that atopic dermatitis of dogs, which is a widely documented risk factor for *M. pachydermatis* infection, would be a risk factor for human carriage. The purpose of this study was to evaluate the prevalence of *M. pachydermatis* in dogs and their owners as determined by microbiologic culture and polymerase chain reaction (PCR). The ultimate goal was to assess whether pet owners could be reservoirs for mechanical transfer of the organism.

## Materials and Methods

### Study Population

Approvals for privately owned animal use and sampling of human participants were obtained from the University of Pennsylvania’s Institutional Animal Care and Use Committee and the biomedical institutional review board, respectively, and informed consent was obtained from participants.

Dogs referred to the Dermatology and Allergy Service of the Matthew J. Ryan Veterinary Hospital of the University of Pennsylvania (VHUP) for evaluation of allergic skin or ear canal disease were screened for secondary *M. pachydermatis* overgrowth (i.e., infection, commonly referred to as malassezia dermatitis or malassezia otitis) by using the tape strip and ear swab methods described below. Dogs with positive cytologic results and their human companions were recruited for the disease group.

A control group of healthy dogs and their human companions were recruited from the faculty, staff, and students at the VHUP. Samples were taken from dogs with normal skin and ear canals (defined as no episodes of skin disease in the preceding calendar year and no evidence of inflammation at the time of sampling) and their human companions by using the same techniques as for the disease group.

### Cytology

The cytologic collection technique used for skin was the tape strip method (*14*). A piece of clear cellophane tape (5 cm x 2 cm) was applied to the surface of the skin 2 times in succession, removed, stained with a modified Wright’s stain (Diff Quik, Dade Behring, Deerfield, IL), and applied to a glass slide for microscopic analysis. From dogs with atopic dermatitis, inflamed skin, which was typically alopecic from self-trauma due to pruritus, was sampled. One or more of the following regions were sampled from each dog: axilla, groin, chin, ventral neck fold, paronychium, and interdigital spaces (dorsal or plantar) according to clinical signs. From dogs with ear canal infections, cotton-tipped swabs of ear canal exudates were collected and streaked onto glass slides, which were then heat fixed and stained.

All slides were examined at 1,000x (high power under oil immersion) magnification. This technique allows for microscopic visualization of any microorganisms that reside on the surface of the skin or within the ear canal cerumen. When a minimum number of yeast cells per oil immersion field (oif) was exceeded (>1 yeast/oif on the skin, >5 yeast/oif in ear canal exudates) (*10*,*15*,*16*), excessive colonization by the organism (i.e., infection) was diagnosed, and these dogs were assigned to the disease group.

### Microbiologic Analysis

For affected dogs, a tape strip was used to sample a positive skin site (an area adjacent to a site positive for yeast), and sterile cotton-tipped swabs were used to sample ear exudates. In healthy control dogs, only the chin and mucocutaneous junction of the lower lip were sampled, since this area is commonly colonized by *M. pachydermatis* (*10*). In the human companions, a single tape strip was used to sample the palms of both hands. To participate, each participant must have abstained from handwashing for at least 1 hour before sampling and must have handled the dog within that period. Veterinary personnel participating in the healthy control group were sampled at least 48 hours after last contact with a veterinary hospital patient.

Tape strips from each pair of participants were placed over drops of sterile olive oil, adjacent to one another, on the surface of a Sabouraud’s dextrose agar plate. The agar was fortified with a drop of olive oil (source of medium- to long-chain fatty acids) to enhance growth of *Malassezia* spp., which are lipophilic (*10*). In cases in which canine ear exudate was sampled rather than skin, the swab was rolled across the surface of the agar incorporating a drop of sterile olive oil.

Plates were incubated at 32°C for up to 7 days. Any fungal colonies isolated were harvested from the tape strips with sterile cotton-tipped swabs and identified cytologically to be yeast by morphologic characteristics. Samples without yeast colonies were discarded. Yeast colonies were then stored at –80°C for future identification of species by polymerase chain reaction (PCR).

### PCR

Samples for PCR were obtained from all dogs and their human companions. For dogs with malassezia dermatitis or otitis, a sterile cotton-tipped swab moistened with sterile saline was used to rub an affected area. For healthy control dogs, the chin and mucocutaneous junction of the lower lip was sampled. For human hands, a sterile gauze pad moistened with sterile saline was used to vigorously rub the hands (palms, fingers, and interdigital webbing) (*17*). Samples were stored in sterile saline at –80°C until used for PCR analysis.

DNA was extracted by using a MasterPure Yeast DNA Purification Kit (Epicentre Technologies, Madison, WI) with the following modifications. The cotton tipped swabs were stored in 1 mL sterile saline. The swabs were brought to room temperature and vortexed briefly. A 200-μL aliquot of saline was then removed from the cryotube and transferred to a sterile 1.5-mL centrifuge tube for DNA extraction. Gauze pads were stored in 10 mL of sterile saline. The pads were also brought to room temperature and were then agitated manually. A 1-mL aliquot was aseptically removed from the bag and transferred to a sterile 1.5-mL centrifuge tube. The tubes were centrifuged for 2 min at 13,000 rpm to pellet all cells, and DNA was extracted as described by the manufacturer.

Species characterization of malassezia DNA was performed by using a nested PCR assay developed by Sugita and colleagues (*18*). Briefly, organisms are identified with species-specific primers derived from the internal transcribed spacer (ITS) region of the ribosomal RNA (rRNA) gene. After amplification of the ITS region, a small aliquot of the reactant is used in a second PCR to identify the *Malassezia* species. The protocol devised by Sugita and colleagues can identify 7 of the currently recognized *Malassezia* species, many of which have been isolated from canine skin. The sensitivity of the assay has been determined by Sugita and colleagues as 1 fg of DNA. As we were specifically interested only in *M. pachydermatis* for the purposes of this study, DNA samples were amplified with *M. pachydermatis*-specific primers. DNA from *M. pachydermatis* ATCC strain 14522 was prepared by American Type Culture Collection and used as a positive control in all reactions.

### Statistical Analysis

To determine differences between culture and PCR in detecting *M. pachydermatis* on humans and dogs, the McNemar test was used. Where applicable, odds ratios (OR) and 95% confidence intervals (CI) were calculated. To determine if the owners of dogs with malassezia dermatitis or otitis were more likely to harbor the yeast than owners of normal dogs, the Fisher exact test was used. Additionally, to assess concordance of culture and PCR results between owner and dog pairs, for both affected and normal groups, the McNemar test was performed. All analyses were performed by using statistical software (StatXact, Version 6, Cytel Software Corp., Cambridge, MA). A p < 0.05 was considered statistically significant.

## Results

**Figure 1:**
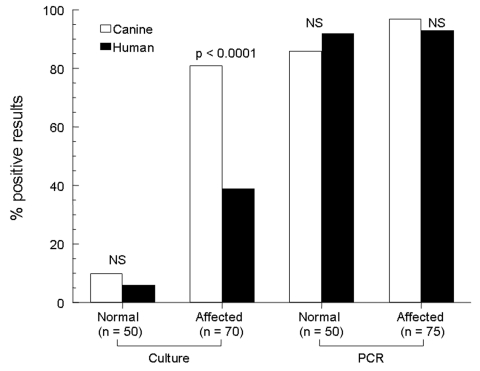
Rates of detection of *Malassezia pachydermatis* on canine and human skin by 2 laboratory techniques. A normal group of dogs and a group known to harbor *M. pachydermatis* infection, paired with their respective owners, are represented. NS, nonsignificant; PCR, polymerase chain reaction.

Fifty healthy dogs and 75 atopic dogs with malassezia dermatitis or otitis and their respective human companions made up the control and affected groups, respectively. Of the control group, 5 (10%) of 50 canine samples were positive for *M. pachydermatis* growth on lipid-enriched Sabouraud’s dextrose agar, and 3 (6%) of 50 human samples were positive (Figure 1). No differences in rates of isolation were seen (p = 0.6).

**Figure 2:**
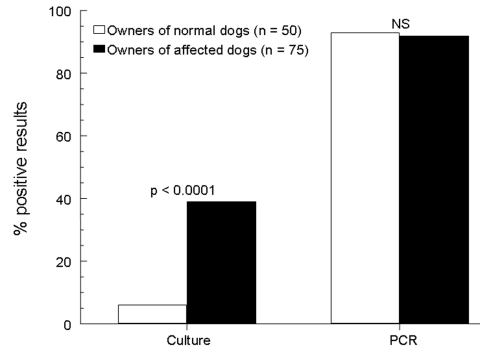
Comparison of rates of detection of *Malassezia pachydermatis* on the hands of dog owners by 2 laboratory techniques. NS, nonsignificant; PCR, polymerase chain reaction.

Of the affected group, 61 (81.3%) of 75 canine samples were positive for *M. pachydermatis*, while 4 samples were overgrown with saprophytic molds before yeast colonies had grown, and 10 were negative. Of the human samples from this group, 29 (38.7%) of 75 were positive, while 5 were overgrown with saprophytic molds, and 41 were negative (Figure 1). For the 70 canine-human pairs with complete culture results, the dogs were more likely to have a positive result than their owners (p < 0.0001). Of the 61 dogs with positive cultures, only 49% had a concordantly positive owner (data for individual pairs not shown). However, all positive owners had dogs that were also positive. When comparing detection of *M. pachydermatis* between owners of normal dogs and owners of affected dogs by culture, the latter were 11.1 times more likely to be positive (95% CI 3.0–59.9, p < 0.0001, Figure 2).

### PCR

Of the control group, 43 (86%) of 50 canine samples and 46 (92%) of 50 human samples were positive for *M. pachydermatis* (Figure 1). All participants (canine or human) with positive culture results were also positive by PCR; however, 38 dogs and 45 humans with negative cultures were positive by PCR. No difference was seen in the rate of detection (p = 0.3) between owner and dog.

Of the affected group, 73 (97.3%) of 75 canine samples and 70 (93.3%) of 75 human samples were positive by PCR for *M. pachydermatis* (Figure 1). Sixty-eight pairs had concordant positive results, and no negative pairs were found. No differences in rates of detection between the dogs and their owners were seen (p = 0.45). When comparing detection of *M. pachydermatis* between owners of affected dogs and owners of normal dogs by PCR, no differences (p = 1.0, 93% vs. 92%, respectively) were seen between groups (Figure 2).

### Microbiology versus PCR

When comparing PCR to culture, regardless of participant species or disease group, PCR was more likely to detect *M. pachydermatis* than culture. For dogs, PCR was 24 times more likely to be positive compared to culture (OR = 24, 95% CI 5.9–98.7), whereas for humans, PCR was 80 times more likely to be positive (OR = 80, 95% CI 11.1–574.9).

## Discussion

Yeast organisms of the genus *Malassezia* are lipophilic fungi that occur as commensal inhabitants of the skin of mammals and birds in very low numbers (*19*). Ten distinct species are now recognized (*20*–*22*), and *M. pachydermatis*, *M. furfur*, *M. globosa*, and *M. sympodialis* are the best characterized with regard to clinical disease correlations (*11*–*13*,*23*,*24*). *M. pachydermatis* is part of the normal cutaneous microflora of dogs and many other mammals (*19*), while *M. furfur*, *M. globosa*, *M. sympodialis*, and *M. restricta* reside naturally on the skin of human beings (*18*,*23*,*25*). Lipophilic organisms exhibit the unique capability of using lipid as a source of carbon. All species except *M. pachydermatis* are entirely lipid dependent. While *M. pachydermatis* does not exhibit an absolute requirement for lipid, its growth is still enhanced by the addition of lipid substrates to culture media (*20*).

In normal dogs with healthy skin, *M. pachydermatis* can routinely be isolated by fungal culture, but proving the presence of the organism by skin surface cytology can be difficult (*10*). In dogs with allergic or seborrheic skin diseases, the homeostasis of the local cutaneous microenvironment is disrupted by inflammation and increased levels of moisture or sebum (*26*,*27*). Under these conditions, the number of *M. pachydermatis* organisms on the skin and in the ear canals may increase dramatically, making it possible to readily identify the organism with rapid cytologic screening (*11*–*13*). Atopic dermatitis may affect up to 10% of the canine population and is the most common reason that dogs are brought to our dermatology clinic for examination. It is also the most common predisposing factor for *M. pachydermatis* infections of the skin and ear canals. The potential for exposure of human beings to the organism is therefore great.

In human beings, especially preterm neonates and immunocompromised adults, *M. furfur* has been shown to cause a systemic bloodborne infection of patients receiving lipid-rich, parenteral nutritional infusions by catheter (*4*). Of zoonotic concern, *M. pachydermatis* has been documented to cause fungemia in similar patient populations (*7*,*9*); however, since this species is not lipid-restricted in its growth, lipid infusion is not a prerequisite for infection (*9*). Chang and colleagues suggested that the source of an outbreak in an intensive care nursery was pet dogs owned by nursing staff who worked in the neonatal intensive care unit (NICU). A single strain of *M. pachydermatis*, as determined by pulsed-field gel electrophoresis, was isolated from infants, the hands of a nurse, and from 3 dogs owned by other healthcare workers in the NICU. This observation suggested that *M. pachydermatis* could represent an emerging zoonotic pathogen.

A limited number of studies have investigated the prevalence of *M. pachydermatis* carriage by human beings. One report identified carriage of very low numbers of the organism on the scalp and palms of 24 (12%) of 200 normal volunteers from whom samples were collected by a washing technique for fungal culture, with subsequent speciation of yeast by biochemical methods (*28*). Although an association with pet ownership was speculated, such information was not collected from the participants. More recent reports have provided much lower estimates of human carriage. One report suggested that *M. pachydermatis* is present on the skin of <1% of normal persons but may be found in ≈2% of dermatitis patients (patients sampled by a swab technique for fungal culture) (*23*), while a second study failed to isolate the organism from either healthy human volunteers or dermatitis patients when application of transparent dressings for subsequent PCR detection was used as the sampling technique (*22*). To date, no single study has directly and systematically addressed the relationship between *M. pachydermatis* carriage on human skin and dog ownership. In the epidemiologic investigation of the NICU outbreak mentioned previously, a total of 53 pets (dogs, cats, and horses) were surveyed, and 12 (31%) of the 39 dogs were positive for *M. pachydermatis*, 3 of which matched the outbreak strain. However, only 1 of 9 nurses, who was not a pet owner, was positive for *M. pachydermatis* (*9*).

The cytologic and microbiologic results from dogs in this study mirror the literature regarding *M. pachydermatis* carriage on the skin of normal and atopic dogs (*10*,*15*,*29*). Ten dogs identified with malassezia infection by cytology were negative on culture. While this finding seems counterintuitive, it is not unusual in our clinical experience. The organism may have failed to grow because of suboptimal culture conditions or nonviable yeast cells. All positive cultures were confirmed to be *M. pachydermatis* by PCR, which confirms our ability to identify the species properly by cytology.

We were significantly less likely to isolate the organism from the skin of normal dogs than from atopic dogs in our study, but this bias was deliberate, since samples for culture were taken from sites that were known to be positive from rapid cytologic screening. However, when PCR was used, no significant difference was seen in detection rates, which reflects the commensal status of the organism on canine skin.

If the culture technique alone had been used, the significantly higher rate of yeast isolation from the hands of the companions of the disease group versus the control group (38.7% vs. 6%) would have supported our hypothesis that active malassezia infection of canine skin or ear canals is a risk factor for human carriage. However, when PCR was used as the detection technique, no significant difference was seen between detection rates on the hands of the 2 human groups (93.3% vs. 94%), which caused us to reject this hypothesis.

As part of the normal microflora of canine skin, *M. pachydermatis* is expected to be detectable by a technique as sensitive as PCR, even from sample sites that do not yield colony growth on culture. With our handwashing and canine skin and ear canal swabbing techniques, a larger surface area was sampled, especially on human hands, and the PCR was presumably able to detect low cell numbers within a large sample aliquot. The culture technique we used for human hands appears to be inadequate for screening purposes; however, we do not know the number or density of viable yeast cells on human hands that may be required to nosocomially spread infection in a clinical setting.

Since reports of *M. pachydermatis*-associated septicemia in humans are relatively scarce, our conclusion is that mechanical carriage of the organism is of low risk to public health. Dogs are commonly used for their therapeutic benefits in clinical settings, such as cancer therapy support groups for children and the elderly, and in psychiatric care facilities. The benefits of canine interaction have been documented (*30*). Advice to pet owners is available at http://www.cdc.gov/healthypets.

In intensive care units, where nosocomial infections are especially problematic, good handwashing practices among healthcare workers are imperative. Unfortunately, little is known about handwashing agents and techniques (e.g., contact time) that will effectively eliminate carriage of malassezia yeast from human hands, and disparate evidence is presented in the literature. In 1 report, improved handwashing practices seemed to eliminate an endemic problem with *M. pachydermatis* infections in a NICU (*9*), while in another, elimination of *M. furfur* from the surfaces of equipment was not achieved with routine hygienic measures (*7*).
